# The Efficacy of the Yasmin[Ethinylestradiol-Drospirenone (0.03/3 mg)] Treatment for Postoperative Abortion: A Protocol of Meta-Analysis

**DOI:** 10.3389/fmed.2022.746668

**Published:** 2022-04-15

**Authors:** Tian Tian, Huiyan Feng, Xiaoqian Liao, Qiling Su, Yunhui Li, Xiaomao Li

**Affiliations:** Department of Gynecology, The Third Affiliated Hospital of Sun Yat-sen University, Guangzhou, China

**Keywords:** Yasmin, antibiotics, motherwort, induced abortion, protocal, meta-analysis

## Abstract

**Background:**

Induced abortion is a universal phenomenon and about 25% of pregnancies in the world end in induced abortion. Induced abortion refers to the use of artificial or drug methods to terminate the pregnancy in the early stage of pregnancy, which is a remedy for failed contraception and accidental pregnancy. Induced abortion means surgical abortion. There are two commonly used methods: negative pressure suction abortion and forceps curettage for induced abortion. Abortion is invasive and it will cause great harm to women's reproductive function. Clinically, there are also cases of re-pregnancy within 3 months after abortion or even re-pregnancy without recovery of menstruation. To improve symptoms and reduce these complications, antibiotics, motherwort, and Yasmin[Ethinylestradiol-Drospirenone (0.03/3 mg)] are clinically used alone or in combination after induced abortion.

**Methods:**

Data were collected from six databases, including three English databases of Cochrane Library, PubMed and Embase, and three Chinese databases of CNKI, Wanfang, and Weipu. The original indicators of vaginal bleeding, menstrual recovery time, bleeding time, endometrial thickness 21 days after surgery and so on were included, and the incidence of postoperative bleeding less than menstrual volume, menstrual re-fluid time ≤ 37 days, bleeding time ≤ 7 days, re-pregnancy without menstruation, re-pregnancy within 3 months after the operation, the total incidence of postoperative complications were based on the ratio of the number of events in the group to the total number of people in the group reported in the literature. Review Manager 5.4 software was downloaded from the Cochrane website to evaluate the quality of the literature and analyze the results using random or fixed-effects models. The outcome of index data is divided into two types, one is dichotomy, and the other is measurement data. The binary data is expressed by odds ratio (OR), and the measurement data is expressed by mean difference (MD), and the confidence interval of both is 95%.

**Results:**

After completing this meta-analysis, the results will be available.

**Conclusion:**

The results will provide reliable data basis for the value of Yasmin combined with antibiotics and Motherwort in postoperative induced abortion.

**PROSPERO Registration Number:**

CRD42021246764.

## Background

Induced abortion is a global phenomenon (1). Approximately 25% of pregnancies around the world end in induced abortion (2). The related data show that the abortion rate in developed countries has dropped significantly since 1990, but not in developing countries. Due to the rapid population growth, the number of induced abortions worldwide reached 50.4 million from 1990 to 1994, and 56.3 million from 2010 to 2014 (2). The abortion rate of women of childbearing age in our country has shown an upward trend since 2014, reaching 28.13‰ in 2016, and decreasing slightly in 2017 (3). The survey shows that in recent years the number of patients undergoing induced abortion in China is about 9 million per year, and the proportion of repeated abortions is also relatively large (4). At present, the rate of induced abortion and repeated abortion in our country is still at a high level, and the first time of sexual behavior and the population receiving induced abortion have shown a trend toward younger age (5).

Induced abortion refers to the use of artificial or drug methods to terminate the pregnancy in the early pregnancy. It is a remedy for failed contraception. It can also be used for those who need to terminate the pregnancy due to diseases that make them unsuitable for continuing pregnancy or to prevent congenital malformations or genetic diseases. There are two methods: surgical abortion and medical abortion, and induced abortion refers to surgical abortion, which has two commonly used methods: negative pressure suction abortion and forceps curettage for induced abortion. Negative pressure suction is the use of a hollow suction tube into the uterine cavity to suck out the embryonic tissue in the uterus through negative pressure, while forceps curettage is to use oval forceps to clamp out the large embryonic tissue in the uterus. Due to the emergence of various painless techniques, and the simple and safe operation, it is widely used in clinical practice.

Induced abortion is invasive and it will do great harm to women's reproductive function.

Bleeding will occur after abortion. This is because under normal circumstances, during the early trimester, hormones in the female body change. The embryo villi planted in the endometrium begin to secrete hormones to support the corpus luteum, which in turn continues to secrete estrogen progesterone to maintain the gradual growth of the endometrium and the normal development of the embryo. The induced abortion removes the embryonic villi rapidly in a short time through surgical methods, and then the hormones secreted by the embryonic villi decline rapidly, and the estrogen and progesterone secreted depending on it also begin to decline. Then the endometrium is peeled off and bleeds due to the loss of the hormone, and the surgery will damage the blood vessels of the endometrium and cause bleeding. Excessive bleeding time and volume are both signs of poor recovery of reproductive function.

Menstrual recovery will occur after abortion. Menstruation refers to the periodical changes that occur in women of normal childbearing age every other month or so, which is characterized by a spontaneous thickening of the endometrium, vascular hyperplasia, gland growth and secretion, and endometrium collapse and shedding. Menstruation stops during normal pregnancy, and abortion is the termination of early pregnancy. After the abortion, the beginning of the next menstrual cycle will start, and menstrual recovery will begin. The sooner the reproductive function is restored, the sooner the menstruation will resume.

The thickness of the endometrium after abortion reflects the recovery of reproductive organs to a certain extent. The endometrial layer refers to the layer that constitutes the inner wall of the mammalian uterus. It responds to both estrogen and progesterone and can change significantly with the menstrual cycle. Abortion causes the termination of pregnancy and the reduction of related hormones. In addition, the surgical operation will damage the endometrium and thin the endometrium, and then the body will gradually recover and the endometrium will gradually thicken.

A variety of complications may occur after abortion, including pelvic infection, intrauterine adhesions, cervical adhesions, intrauterine residuals, menstrual disorders, amenorrhea, and so on.

Pelvic infection often occurs after abortion. As abortion will destroy the physiological defense mechanism, the body's immunity will decrease, resulting in postoperative pelvic infection, which is one of the common postoperative complications.

Induced abortion may cause intrauterine adhesions. The stimulation of induced abortion operation can damage the endometrium, and then cause pathological changes of the endometrium, including the destruction of the basal layer of the endometrium, the exudation and deposition of fibrinogen in the uterine stroma, which leads to adhesion of the front and back walls of the uterine cavity. The occurrence of intrauterine adhesions involves a large number of cytokines and complex interactions between cell factors (6). The essence of intrauterine adhesions is endometrial fibrosis. Many scholars at home and abroad believe that any damage of endometrial can lead to the occurrence of intrauterine adhesions (7). Uterine adhesion is a common complication after induced abortion, with an incidence rate of 10 to 20% (8).

Abortion may also cause cervical adhesions. The operation of abortion requires a curettage or suction operation. On the one hand, in order to avoid residual intrauterine, it needs to be repeated many times and the operation time is prolonged, thereby aggravating the damage to the endocervical endometrium, endometrium, etc.; on the other hand, the improper setting of parameters in uterine operation may cause excessive damage to the endocervical endometrium and uterine lining due to excessive negative pressure. Moreover, since The endometrium and cervical canal are both relatively thin, these improper operations may lead to damage in deep parts, and then the functional layer is replaced by a single layer of epithelial cells during the recovery process, forming fibrotic adhesions. In addition, when combined infection occurs after surgery, under the impact of bacterial toxins and related enzymes, the endocervical endometrium and uterine lining will necrosis, forming necrotic granulomas, and then cause the inflammation responds, such as the deposition of hemosiderin, dark brown carbon material, and calcification, etc., then causing adhesions between the cervix and the uterine cavity (9).

Intrauterine residues may occur after abortion. Residues in the uterus are caused by incomplete suction or missed suction, and it is a common complication after artificial abortion. The occurrence of it is related to the abnormal position of the patient's uterus, the lack of clinical experience of the doctor, and the patient's repeated uterine cavity operations in the past. Residues in the uterus will lead to prolonged vaginal bleeding after surgery and increase the chance of infection.

Induced abortion may cause serious long-term fertility damage, such as postoperative amenorrhea, one of the common long-term complications, which refers to more than 3 cycles of amenorrhea after induced abortion. There are three main reasons for postoperative amenorrhea. One is the adhesion of the cervical canal. The inner lining of the cervical canal is damaged, which leads to the adhesion of the cervical canal. When it is completely closed, it will also show amenorrhea. The second is the adhesion of the uterine cavity. Irreparable damage or serious infection of the membrane has caused the front and back walls of the uterus to adhere, resulting in amenorrhea. The third is reproductive endocrine disorders. After an abortion, women will be emotionally nervous, fearful, sad, etc., which will cause endocrine disorders and cause amenorrhea (10).

Menstrual disorders may occur after abortion. Some scholars believe that improper techniques such as excessive suction in induced abortion can cause damage to the blood vessels of the endometrium. As there are abundant estrogen and progesterone receptors in the endometrium, improper abortion techniques will damage the inner uterus. In this case, even if the level of estrogen and progesterone is normal, the normal growth of the endometrium cannot be guaranteed, resulting in menstrual disorders after surgery. There are also scholars of traditional Chinese medicine who believe that induced abortion can cause Qi and blood damage, coupled with feelings of depression, external evil accumulation, and other factors that can lead to the formation of blood stasis, resulting in kidney deficiency and blood stasis, leading to menstrual disorders (11).

There may be another pregnancy within 3 months after induced abortion or even a second pregnancy without menstruation. Ovulation can be resumed 2–3 weeks after the abortion, and ovulation can occur as early as 11 days after the operation, which will cause another pregnancy without recovery of menstruation and cause another miscarriage. Repeated abortion refers to 2 or more induced abortions, which will cause greater harm to women's bodies. Repeated abortion within 1 year, especially within 6 months, is the most harmful, which has an adverse effect on long-term fertility and future pregnancy outcomes. In addition, the failure to take effective contraception after the operation that leads to another abortion within 3 months greatly increases the incidence of various complications. Therefore, it is particularly important to take contraceptive measures immediately after abortion.

Some studies have also pointed out that induced abortion is an important cause of secondary infertility (12). In addition, it will also have a great impact on women's psychology. A study has shown that induced abortion is significantly related to depression and adjustment disorders, and there is a positive correlation between induced abortion and some mental diseases (13). More studies have shown that among Chinese women, a higher number of pregnancies and miscarriages are associated with a higher risk of diabetes (14). Another study showed that a high risk of cardiovascular disease is associated with both induced abortion and re-abortion among Chinese women (15). An early study in Europe showed that there is a significant correlation between induced abortion and premature birth, and the risk of premature birth increases with the increase of the number of induced abortion (16).

In order to alleviate symptoms and reduce these complications, antibiotics, motherwort, and Yasmin[Ethinylestradiol-Drospirenone (0.03/3 mg)] are clinically used alone or in combination after induced abortion. However, how to use them has different clinical opinions and many studies. In fact, Yasmin[Ethinylestradiol-Drospirenone (0.03/3 mg)] combined with antibiotics and motherwort are more common.

Clinically, antibiotics are often used after abortion to fight infection and Leonurus to promote uterine contraction (42). Induced abortion is an invasive procedure, and the latest ACOG guidelines suggest that women with induced abortion should use prophylactic antibiotics (43). A study showed that in obstetrics and gynecology surgery, the use of antibiotics can effectively reduce postoperative infection rate and postoperative lesion rate (44)^.^ Studies have shown that basic antibiotics such as metronidazole, amoxicillin, and azithromycin are feasible for the prevention of infection after an abortion (17). More early studies have shown that the most cost-effective way to reduce the risk of infection may be the universal use of antibiotic prophylaxis (18). Leonurus is a kind of Chinese medicine, which comes from the fresh or dry ground parts of Leonurus belonging to labiatae. Leonurus contains a variety of chemical components, more than 120 compounds have been isolated from Motherwort, including alkaloids, diterpenes, flavonoids, phenylethanol glycosides, phenylpropanin, coumarin, triterpenes, organic acids, volatile oil and other components. Research has proved that Leonurus has the capability of two-way regulation for uterine contraction activities, namely when the uterus is in a normal state, Leonurus can cause uterine contractions, and when the uterus is in a state of spasm, Leonurus has a relaxing effect on it, and also regulates uterine myoelectricity, protects the endometrium and promotes endometrial involution, anti-endometritis etc. (19, 38). Leonurus has a bidirectional regulation effect on uterus. Pharmacological studies have shown that water-soluble alkaloids and total flavonoids of Leonurus have an excitatory effect on the uterus *in vitro*, the mechanism of which is related to increasing the cytoplasmic Ca2+ content of uterine smooth muscle cells, and the fat soluble alkaloids have a certain inhibitory effect on the uterus *in vitro* (39). The water extract of Leonurus can excite the uterus of normal rats *in vitro* and inhibit the uterus excitation induced by oxytocin (40). Leonurus accelerates postpartum uterine recovery, the mechanism may be that Leonurus reduces the TNF-α level of postpartum inflammatory uterus, down-regulates the expression of TIMP-1, starts the hemostatic repair mechanism, and accelerates the degradation of extracellular matrix (ECM), thus accelerating postpartum uterine irecovery (41). Studies have also shown that Motherwort can regulate uterine contractions in both directions, regulate uterine myoelectricity, protect the endometrium, promote endometrial involution, and resist endometritis (19).

Yasmin[Ethinylestradiol-Drospirenone (0.03/3 mg)], which is a new low-dose single-phase oral short-acting contraceptive, is a compound preparation of Ethinyl Estradiol and drospirenone (20), each containing Ethinyl Estradiol 3 mg and drospirenone 0.03 mg. Yasmin[Ethinylestradiol-Drospirenone (0.03/3 mg)] is now commonly used after an induced abortion (21). Drospirenone in Yasmin[Ethinylestradiol-Drospirenone (0.03/3 mg)] is a new synthetic progestin with antimineralocorticoid activity and can be used for effective contraception. It also has other beneficial properties: first, it has antimineralocorticoid activity, which can prevent weight gain and other symptoms caused by body fluid retention; second, it has antiandrogen activity, has a good effect on the skin, and can reduce acne damage and sebum. Third, it does not have any androgen, estrogen, glucocorticoid, and anti-glucocorticoid activity. The combination of these three characteristics makes the biochemical and pharmacological properties of drospirenone very similar to natural progesterone. Yasmin[Ethinylestradiol-Drospirenone (0.03/3 mg)] is rapidly absorbed orally, and its half-life of drospirenone is 30 to 35h. It can reach a steady-state concentration in plasma after 10 days of treatment. About 95% of drospirenone combines with plasma albumin, and 5% exists in free form in plasma. Its metabolites are mainly excreted by the kidneys (20). Serum Ethinyl Estradiol levels show a biphasic decline, and its half-lives are about 1 h and 10–20 h, respectively. It is not excreted as a prototype. Its metabolites are excreted in urine and bile and can reach a steady state in the second half of a treatment cycle. Studies have found that after an abortion, the use of Yasmin[Ethinylestradiol-Drospirenone (0.03/3 mg)] for treatment has a considerable effect, which not only can effectively help repair the endometrium and alleviate vaginal bleeding but also is quite safe and can be clinically promoted (22). The use of short-acting oral contraceptives Yasmin[Ethinylestradiol-Drospirenone (0.03/3 mg)] after abortion can reduce vaginal bleeding volume and bleeding time and promote menstrual recovery, which is the core content of family planning services after an induced abortion (23).

Yasmin[Ethinylestradiol-Drospirenone (0.03/3 mg)] is effective in contraception. The contraceptive mechanism of Yasmin[Ethinylestradiol-Drospirenone (0.03/3 mg)] is mainly through feedback inhibition of gonadotropins to inhibit ovulation, changing cervical mucus to make it difficult for sperm to pass, and at the same time acting on the endometrium to interfere with embryo implantation. If the contraceptives such as Yasmin[Ethinylestradiol-Drospirenone (0.03/3 mg)] are used correctly, the contraceptive effect can reach more than 99% (24).

Yasmin[Ethinylestradiol-Drospirenone (0.03/3 mg)] can shorten the time of menstrual recovery. The progesterone drospirenone in Yasmin[Ethinylestradiol-Drospirenone (0.03/3 mg)] can change the endometrium from the proliferative phase to the secretory phase. After stopping the drug, the endometrium falls off, which promotes the recovery of menstruation and shortens the time of menstrual resumption (25).

Yasmin[Ethinylestradiol-Drospirenone (0.03/3 mg)] can shorten the bleeding time, reduce the amount of vaginal bleeding, and increase the thickness of the endometrium. Studies have shown that taking Yasmin[Ethinylestradiol-Drospirenone (0.03/3 mg)] immediately after induced abortion can promote endometrial repair, increase endometrial thickness, reduce vaginal bleeding volume, and shorten bleeding time (26, 27). And Yasmin[Ethinylestradiol-Drospirenone (0.03/3 mg)] has more advantages over other compound oral contraceptives in protecting the endometrium of patients after an abortion (28).

Oral Yasmin[Ethinylestradiol-Drospirenone (0.03/3 mg)] after artificial abortion can effectively prevent and reduce complications (29).

Yasmin[Ethinylestradiol-Drospirenone (0.03/3 mg)] can reduce residues in the uterus. The estrogen in Yasmin[Ethinylestradiol-Drospirenone (0.03/3 mg)] can speed up the repair of the endometrium, prevent bleeding, especially help the endometrium adhered to the residue on the endometrium to repair quickly, accelerate the separation of the residue on the uterus, and promote their removal as soon as possible (30).

Yasmin[Ethinylestradiol-Drospirenone (0.03/3 mg)] can reduce intrauterine adhesions. Yasmin[Ethinylestradiol-Drospirenone (0.03/3 mg)] can effectively prevent intrauterine adhesions (31, 32). Ethinylestradiol in Yasmin[Ethinylestradiol-Drospirenone (0.03/3 mg)] can increase uterine blood supply, increase endometrial glands, stroma, and blood vessels, thus promoting rapid endometrial hyperplasia and timely repair, terminating abnormal uterine bleeding, protecting endometrial basal layer and its functions, and reducing intrauterine adhesions (25, 33).

Yasmin[Ethinylestradiol-Drospirenone (0.03/3 mg)] can reduce cervical adhesions and reduce postoperative amenorrhea. Studies have shown that postoperative use of Yasmin[Ethinylestradiol-Drospirenone (0.03/3 mg)] can significantly reduce the incidence of postoperative cervical adhesions and amenorrhea (26).

Yasmin[Ethinylestradiol-Drospirenone (0.03/3 mg)] can reduce postoperative pelvic infections. Induced abortion combined with Yasmin[Ethinylestradiol-Drospirenone (0.03/3 mg)] treatment can better alleviate the vaginal bleeding of aborted women, reduce postoperative complications of pelvic infection, and improve the clinical effect (34).

Yasmin[Ethinylestradiol-Drospirenone (0.03/3 mg)] can reduce the incidence of postoperative menstrual disorders. The Ethinyl Estradiol in Yasmin[Ethinylestradiol-Drospirenone (0.03/3 mg)] can repair the hyperplasia of the endometrium damaged by over-absorption, improve the clinical symptoms, and is often used for the prevention and treatment of postoperative irregular menstruation (11, 35).

There have been numerous studies on the value of Yasmin[Ethinylestradiol-Drospirenone (0.03/3 mg)] used immediately after abortion, but there's only one literature on the systematic analysis of Yasmin[Ethinylestradiol-Drospirenone (0.03/3 mg)] published in 2014, and the included indicators are only postoperative vaginal bleeding time and bleeding volume, postoperative endometrial thickness, and postoperative menstrual recovery time. However, there are many clinical studies on the reduction of postoperative complications after abortion. Therefore, it is necessary to comprehensively analyze the use of Yasmin[Ethinylestradiol-Drospirenone (0.03/3 mg)] after abortion.

## Methods

### Study Registration

This protocol was registered with the International Platform of Registered Systematic Review and Meta-Analysis Protocols (PROSPERO) (registration number CRD42021246764).

### Inclusion Criteria for Study Selection

#### Types of Studies

Randomized controlled clinical trials involving the use of Yasmin[Ethinylestradiol-Drospirenone (0.03/3 mg)] after induced abortion, and they are not limited by language.

#### Types of Participants

The subjects are patients after induced abortion, and the surgical methods are all included regardless of whether ultrasound is used.

#### Types of Interventions

##### Test Group

The experimental group is Yasmin[Ethinylestradiol-Drospirenone (0.03/3 mg)] combined with antibiotics and Leonuri.

##### Control Group

The control group is treated with antibiotics and motherwort.

### Outcome Indicators

The original indicators of vaginal bleeding, menstrual recovery time, bleeding time, endometrial thickness, and so on in 21 days after surgery are included, and the incidence of postoperative bleeding less than menstrual volume, menstrual re-fluid time ≤ 37 days, bleeding time ≤ 7 days, re-pregnancy without menstruation, re-pregnancy within 3 months after the operation, the total incidence of postoperative complications, menstrual disorders, intrauterine residual, amenorrhea, intrauterine adhesions, cervical adhesions, and pelvic infections are all based on the ratio of the number of events to the total number of people in the group in the literature.

### Exclusion Criteria

(1) The research subjects do not match the interventions; (2) the primary intervention is Yasmin, and the control groups are multiple or other types, such as the one using contraceptive rings; (3) The same treatment for the intervention and control is not antibiotics combined with motherworts; and (4) the types of literature are nursing and crossover trials.

### Database and Retrieval Strategies

Six major databases were searched on the Internet, including Cochrane Library, PubMed, Embase, CNKI, Wanfang, and Vip, and randomized controlled trials published before October 23, 2020, were searched without any language restrictions. We searched the literature by a combination of subject and free terms, including disease and intervention, and type of study. Take PubMed as an example, the specific search strategy is shown in Table 1. After all pieces of literature are searched, RCT and deadline date are selected.

**Table 1:** PubMed retrieval strategies.

**ID**	**Query**
#1	“Abortion, Induced” [Mesh]
#2	((((((((((((((((((((((((((((((((((((((((((((((((((((((((((((((((Induced Abortion[Title/Abstract]) OR (Abortions, Induced[Title/Abstract])) OR (Induced Abortions[Title/Abstract]))) OR (Abortion (Induced)[Title/Abstract])) OR (Abortions (Induced)[Title/Abstract])) OR (Abortion Rate[Title/Abstract])) OR (Abortion Rates[Title/Abstract])) OR (Rate, Abortion[Title/Abstract])) OR (Rates, Abortion[Title/Abstract])) OR (Abortion Techniques[Title/Abstract])) OR (Abortion Technique[Title/Abstract])) OR (Technique, Abortion[Title/Abstract])) OR (Techniques, Abortion[Title/Abstract]))) OR (Abortion Technics[Title/Abstract])) OR (Abortion Technic[Title/Abstract])) OR (Technic, Abortion[Title/Abstract])) OR (Technics, Abortion[Title/Abstract])) OR (Abortion, Drug-Induced[Title/Abstract]))) OR (Abortion, Drug Induced[Title/Abstract])) OR (Abortions, Drug-Induced[Title/Abstract])) OR (Drug-Induced Abortion[Title/Abstract])) OR (Drug-Induced Abortions[Title/Abstract])) OR (Previous Abortion[Title/Abstract])) OR (Abortion, Previous[Title/Abstract])) OR (Abortions, Previous[Title/Abstract])) OR (Previous Abortions[Title/Abstract])) OR (Abortion History[Title/Abstract])) OR (Abortion Histories[Title/Abstract])) OR (Histories, Abortion[Title/Abstract])) OR (History, Abortion[Title/Abstract])) OR (Abortion, Saline-Solution[Title/Abstract])) OR (Abortion, Saline Solution[Title/Abstract])) OR (Abortions, Saline-Solution[Title/Abstract])) OR (Saline-Solution Abortion[Title/Abstract])) OR (Saline-Solution Abortions[Title/Abstract])) OR (Abortion, Soap-Solution[Title/Abstract])) OR (Abortion, Soap Solution[Title/Abstract]))) OR (Abortions, Soap-Solution[Title/Abstract])) OR (Soap-Solution Abortion[Title/Abstract])) OR (Soap-Solution Abortions[Title/Abstract])) OR (Anti-Abortion Groups[Title/Abstract]))) OR (Anti Abortion Groups[Title/Abstract])) OR (Anti Abortion Groups[Title/Abstract])) OR (Group, Anti-Abortion[Title/Abstract])) OR (Groups, Anti-Abortion[Title/Abstract])) OR (Embryotomy[Title/Abstract]))) OR (Embryotomies[Title/Abstract])) OR (Fertility Control, Postconception[Title/Abstract])) OR (Postconception Fertility Control[Title/Abstract])) OR (Abortion Failure[Title/Abstract])) OR (Abortion Failures[Title/Abstract])) OR (Failure, Abortion[Title/Abstract])) OR (Failures, Abortion[Title/Abstract])) OR (Abortion, Rivanol[Title/Abstract]))) OR (Abortions, Rivanol[Title/Abstract]))) OR (Rivanol Abortion[Title/Abstract])) OR (Rivanol Abortions[Title/Abstract])
#3	#1 OR #2
#4	“Ethinyl Estradiol” [Mesh]
#5	((((((((((((((((((Estradiol, Ethinyl[Title/Abstract]) OR (Ethynyl Estradiol[Title/Abstract])) OR (Estradiol, Ethynyl[Title/Abstract])) OR (Ethinyloestradiol[Title/Abstract])) OR (19-Norpregna-1,3,5(10)-trien-20-yne-3,17-diol, (17alpha)- Ethinyl Estradiol Hemihydrate[Title/Abstract])) OR (Hemihydrate, Ethinyl Estradiol[Title/Abstract])) OR (Ethinyl Estradiol, (8 alpha)-Isomer[Title/Abstract])) OR (Ethinyl Estradiol, (8 alpha,17 alpha)-Isomer[Title/Abstract])) OR (Ethinyl Estradiol, (8 alpha,9 beta,13 alpha,14 beta)-Isomer[Title/Abstract])) OR (Progynon C[Title/Abstract])) OR (Microfollin[Title/Abstract])) OR (Microfollin Forte[Title/Abstract])) OR (Ethinyl-Oestradiol Effik[Title/Abstract])) OR (Ethinyl Oestradiol Effik[Title/Abstract])) OR (Ethinylestradiol Jenapharm[Title/Abstract])) OR (Jenapharm, Ethinylestradiol[Title/Abstract])) OR (Lynoral[Title/Abstract])) OR (Estinyl[Title/Abstract])) OR (Ethinyl Estradiol, (9 beta,17 alpha)-Isomer[Title/Abstract])
#6	#4 OR #5
#7	#3 AND #6

*Mesh, medical subject headings*.

### Data Extraction and Management

#### Literature Inclusion

**Figure 1:**
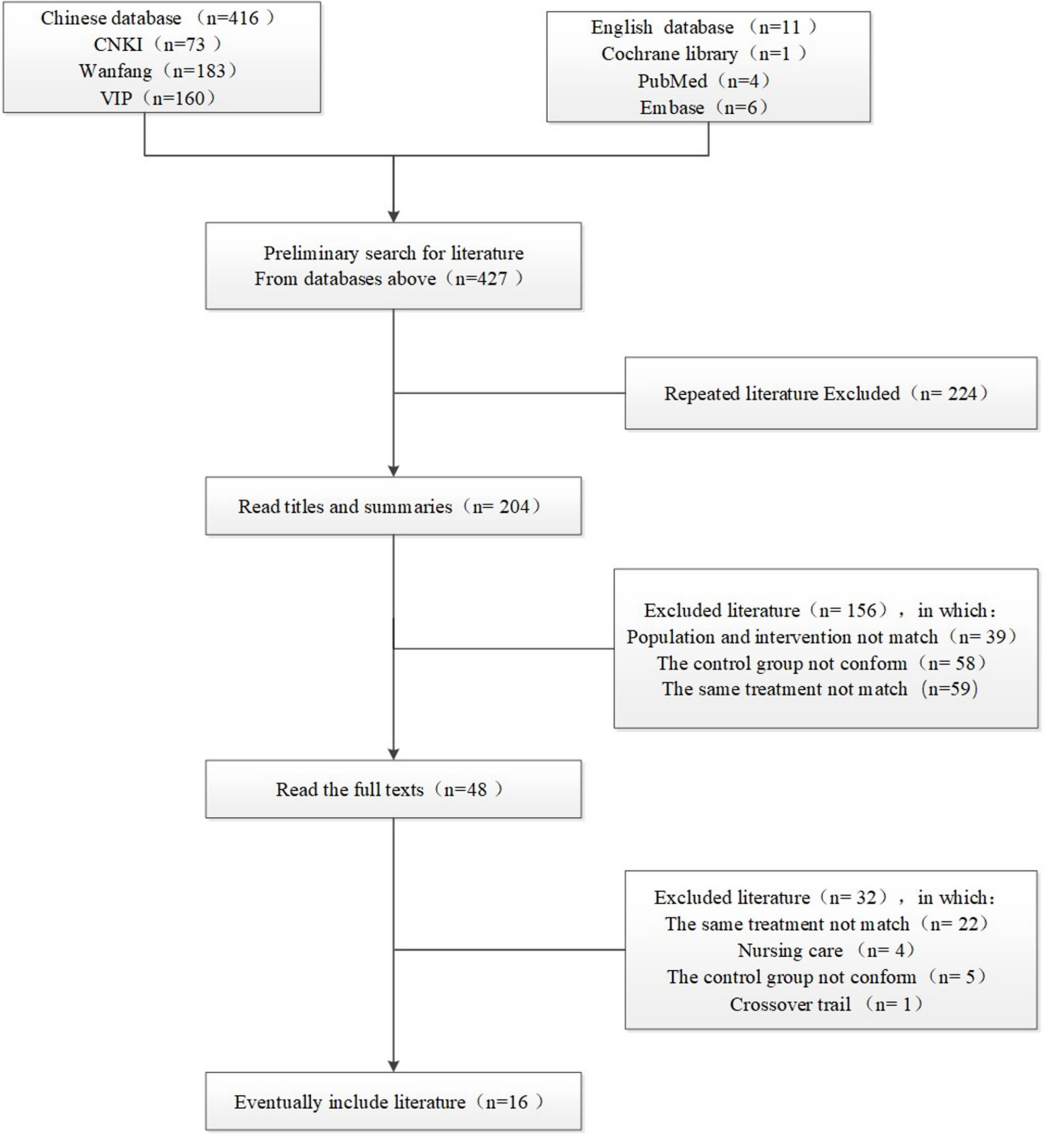
Literature screening flow chart.

Six databases were searched on the Internet, and the required documents were found in the first place. Then the duplicate pieces of literature were eliminated, and then those that were not in conformity were eliminated through reading the title and abstract, and then the full text was downloaded and read, and then excluded again, and finally include all the remaining required. The search flow chart is shown in Figure 1.

#### Data Extraction

Data were extracted independently from the included literature by 2 investigators and reviewed by a third investigator.Basic data information included the name of the first author, year, sample size, gender, course of disease, age, intervention, course of treatment, adverse response, outcome indicators, and baseline comparison before treatment.

#### Methodological Quality Assessment

Review Manager5.4 software was used for entry, including 6 programs: random grouping method, allocation concealment, Blinding of personnel, Blinding of participants, follow-up/case integrity, and outcome indicator integrity. Each program is divided into three levels: “low risk”, “unknown risk” and “high risk”. The scoring criteria are: low risk is 1 point, high risk or unknown risk is 0 point. The scores of each item in each literature were added up, and 5–6 was classified as low risk, 3–4 as unknown risk, and 1–2 as high risk.

### Statistical Analysis

#### Quantitative Data Synthesis

RevMan 5.4 software was used for data analysis. Outcome index data can be divided into two types, one is dichotomy, the other is measurement data. The outcome indicators including incidence of postoperative bleeding less than menstrual volume, menstrual re-fluid time ≤ 37 days, bleeding time ≤ 7 days, re-pregnancy without menstruation, re-pregnancy within 3 months after the operation, the total incidence of postoperative complications, menstrual disorders, intrauterine residual, amenorrhea, intrauterine adhesions, cervical adhesions, and pelvic infections are dichotomous data, and they are presented by odds ratio (OR). And the outcome indicators, such as vaginal bleeding, menstrual recovery time, bleeding time, and endometrial thickness 21 days after surgery were quantitative data, and they are presented by the mean difference (MD) with confidence intervals of 95% (95% CI).

#### Assessment of Heterogeneity

A heterogeneity test was performed first in all studies, and the i2 test was used. When *P* < 0.1 or i2 > 50%, the heterogeneity test was considered to have significant differences, and the random-effect model was used. Conversely, when *P* > 0.1 or i2 < 50%, a fixed-effect model was used.

#### Publication Bias

When the number of qualified literature was sufficient, funnel plots were used to check for publication bias.

#### Subgroup Analysis

Subgroup analysis was determined according to the type of outcome indicators, such as postoperative complications, and subgroup analysis was performed according to the specific type of complications.

## Discussion

Induced abortion refers to the operation to terminate the pregnancy in the early trimester, and it is traumatic and will cause great harm to females. It takes a certain time for the function of female reproductive organs to recover after abortion, and it may also cause a variety of postoperative complications, such as pelvic infection, intrauterine adhesions, cervical adhesions, residual intrauterine, menstrual disorders, amenorrhea, etc. Non-contraception and contraceptive failure are the main reasons for abortion. Ovulation can be restored 2 weeks after an abortion, and the relevant guidelines recommend that contraceptive measures should be taken immediately after abortion.

There are three main methods of contraception after induced abortion.

The first is the intrauterine device, which is a long-acting contraceptive measure. It is suitable for women with poor compliance who need longer-term contraception. The immediate placement of IUDs after induced abortion should first ensure that the operation is thorough and excessive curettage is not allowed. At the same time of anti-infective treatment, drugs for hemostasis and regulating menstruation should be used for comprehensive treatment. Placement should be terminated for patients with serious complications (20).

The second is barrier contraception. Male condoms (i.e. penile condoms) are a commonly used method of barrier contraception. The correct use of contraception can be effective, but there are many problems such as affecting the quality of sexual life, failure to keep using condoms every time, and failure to use correctly, and so on.

The third is short-acting oral contraceptives, which are highly effective and safe (20). Currently, the commonly used tablet is Yasmin[Ethinylestradiol-Drospirenone (0.03/3 mg)], which are a combination of drospirone and ethinylestradiol (20). Each tablet contains 3 mg of ethinylestradiol and 0.03 mg of drospirenostrone.

Yasmin[Ethinylestradiol-Drospirenone (0.03/3 mg)] is commonly used after abortion. Drospirenone in Yasmin[Ethinylestradiol-Drospirenone (0.03/3 mg)] is a new synthetic progesterone with antimineralocorticoid activity. Its biochemical and pharmacological properties are very similar to those of natural progesterone, and it is highly effective in contraception. A large number of studies also believe that Yasmin[Ethinylestradiol-Drospirenone (0.03/3 mg)] can speed up postoperative body function recovery, reduce postoperative bleeding time, promote postoperative menstrual re-moistness, reduce vaginal bleeding volume, speed up endometrial repair, increase endometrial thickness, and reduce postoperative abortion Complications, etc. (36, 37).

There have been numerous studies on the value of Yasmin[Ethinylestradiol-Drospirenone (0.03/3 mg)] used immediately after abortion, but there's only one literature on the systematic analysis of Yasmin[Ethinylestradiol-Drospirenone (0.03/3 mg)] published in 2014, and the included indicators are only postoperative vaginal bleeding time and bleeding volume, postoperative endometrial thickness, and postoperative menstrual recovery time. However, there are many clinical studies on the reduction of postoperative complications after abortion. Therefore, it is necessary to comprehensively analyze the use of Yasmin[Ethinylestradiol-Drospirenone (0.03/3 mg)] after abortion.

The results of the hypothesis show that, first, the use of Yasmin[Ethinylestradiol-Drospirenone (0.03/3 mg)] immediately after abortion can adjust the menstrual cycle, so that the female body quickly recover. It can increase the incidence of less bleeding than menstruation, shorten the time of menstruation revulsion, increase the incidence of menstruation revulsion time ≤ 37, shorten the postoperative bleeding time, increase the incidence of bleeding time ≤ 7 days, and increase the endometrial thickness 21 days after surgery. This may be due to Yasmin[Ethinylestradiol-Drospirenone (0.03/3 mg)], a new low-dose single-phase oral short-acting contraceptive that is a combination of ethinylestradiol and drospirone (20), each containing 3 mg ethinylestradiol and 0.03 mg drospirone. Drospirenone in Yasmin[Ethinylestradiol-Drospirenone (0.03/3 mg)] is a novel synthetic progesterone with anti-salt corticosteroid activity, which is highly effective in contraception. It also has other beneficial properties. First, it has anti-salt corticosteroid activity, which prevents weight gain and other symptoms caused by fluid retention. Second, it has anti-androgen activity, has a good effect on the skin, can reduce acne damage and sebum production; Third, there is no androgen, estrogen, glucocorticoid and anti-glucocorticoid activity. These three properties combine to make the biochemical and pharmacological properties of drospirosterone very similar to those of natural progesterone. The progesterone drospirosterone in Yasmin[Ethinylestradiol-Drospirenone (0.03/3 mg)] can change the endometrium from the hyperplasia stage to the secretion stage, and the endometrium falls off after withdrawal, promoting the recovery of menstruation (25). However, there was no significant effect on vaginal bleeding, which may be related to individual differences and regional factors.

Second, the immediate use of Yasmin[Ethinylestradiol-Drospirenone (0.03/3 mg)] after abortion can reduce the incidence of re-pregnancy after menstrual failure and the incidence of re-pregnancy within 3 months after surgery. The reason may be that drospirenone and ethinyl estradiol tablets are effective contraceptives. Its contraceptive mechanism is mainly through feedback inhibition of gonadotropins to inhibit ovulation, change cervical mucus to make sperm difficult to pass, role in embryo implantation endometrial and interference at the same time, and some studies have found that Yasmin[Ethinylestradiol-Drospirenone (0.03/3 mg)] contraceptive effect can reach more than 99% after proper use (24).

Third, the immediate use of Yasmine[Ethinylestradiol-Drospirenone (0.03/3 mg)] after abortion can reduce postoperative complications (including the incidence of total complications, menstrual disorders, intrauterine residues, amenorrhea, intrauterine adhesion, cervical adhesion, pelvic infection, etc.). It may because ethinylestradiol in Yasmine can repair endometrium hyperplasia and improve clinical symptoms, and it is often used for the prevention and treatment of postoperative menstrual irregularities (11, 35). Estrogen in Yasmin[Ethinylestradiol-Drospirenone (0.03/3 mg)] can speed up the repair of endometrium and prevent bleeding, especially for the rapid repair of the endometrium adhered to the residues on the endometrium, accelerate the separation of residues on the uterus and promote its excretion as soon as possible (30). Ethinyl estradiol in Yasmin[Ethinylestradiol-Drospirenone (0.03/3 mg)] can increase uterine blood flow, increase endometrial glands, stroma and blood vessels, thus promoting rapid endometrial hyperplasia and timely repair, terminating abnormal uterine bleeding, protecting endometrial basal layer and its function, and reducing intrauterine adhesions (25, 33). Induced abortion combined with the treatment of Yasmin[Ethinylestradiol-Drospirenone (0.03/3 mg)] can better improve the vaginal bleeding of women with abortion, reduce the incidence of postoperative pelvic infection complications, and improve the clinical effect (34).

However, the hypothesis may not be valid, and the possible reasons are as follows: first, the quality of the literatures included in the meta-analysis is different; second, the literatures have certain heterogeneity, such as different treatment courses of Yasmin[Ethinylestradiol-Drospirenone (0.03/3 mg)]; third, there is bias.

## Author Contributions

XLi performed critical revision of this manuscript. TT conceptualized the research idea, developed the research design and drafted the manuscript. HF registered the protocol and modified the manuscript. XLia, QS, and YL contributed to the study design. All authors read and approved the final manuscript.

## Conflict of Interest

The authors declare that the research was conducted in the absence of any commercial or financial relationships that could be construed as a potential conflict of interest.

## Publisher's Note

All claims expressed in this article are solely those of the authors and do not necessarily represent those of their affiliated organizations, or those of the publisher, the editors and the reviewers. Any product that may be evaluated in this article, or claim that may be made by its manufacturer, is not guaranteed or endorsed by the publisher.

## Abbreviations

CI: confidence interval
OR: odds ratio
MD: average difference
RCT: randomized controlled trial.
